# Arrhythmias and the extracardiac conduit Fontan: promise unfulfilled?

**DOI:** 10.1093/europace/euae099

**Published:** 2024-04-23

**Authors:** Seshadri Balaji, Susan P Etheridge

**Affiliations:** Department of Pediatrics, Division of Cardiology, Oregon Health & Science University and Boise St. Luke’s Medical Center, 600 East Jefferson Ave, Boise ID 83712, USA; Department of Pediatrics, Division of Cardiology, Oregon Health & Science University and Boise St. Luke’s Medical Center, 600 East Jefferson Ave, Boise ID 83712, USA

**This editorial refers to ‘Long-term incidence of arrhythmias in extracardiac conduit Fontan and comparison between systemic left and right ventricle’ by C. Di Mambro *et al*., https://doi.org/10.1093/europace/euae097.**

A few decades ago, survival of the single ventricle patient to adulthood was rare. The Fontan procedure, performed for palliation of this population, remains in use but has had numerous modifications since its inception in 1971.^1,2^ Fontan surgery separates the systemic from pulmonary circulation by directing systemic venous return to the pulmonary artery without an interposed sub-pulmonary ventricle.^2^ Arrhythmias are known to plague this population despite many modifications to the surgical technique.^2^ In 1988, de Leval *et al.*^3^ reported the total cavo-pulmonary connection modification, which now goes by the term intracardiac lateral tunnel (ILT). Soon after, in 1990, Marcelletti *et al.*^4^ from Ospedale ‘Bambino Gesu’ in Rome described a modification to de Leval *et al.*’s idea, namely the extracardiac conduit (ECC) in four patients. The paediatric cardiac surgery community has largely adopted the ECC as the preferred way to perform the Fontan.^5^ In this issue of *Europace*, Di Mambro *et al.*^6^ outline the long-term arrhythmia issues in an older ECC Fontan population, likely the oldest available as these researchers originate from the same Bambino Gesu Hospital where the first ECC Fontan procedures were performed.^6^

In the original 1990 manuscript, Marcelletti *et al.*^4^ speculated that the ECC Fontan would reduce the incidence of supraventricular arrhythmias by avoiding intra-atrial scars and prosthetic material. Over the next decade, while waiting for this new Fontan population to age, cardiologists and surgeons speculated that the ECC Fontan would decrease the incidence of atrial arrhythmias by minimizing factors that predispose patients to arrhythmia generation, namely, elevated right atrial pressures, atrial incisions and suture lines, surgery near the sinus node or its blood supply, and ventricular dysfunction resulting from ischaemic arrest and long cardiopulmonary bypass times.^7^ Multiple studies have attempted to compare the incidence of arrhythmias between the ILT and the ECC with mixed results.^8–10^ While the ECC appeared to be associated with fewer arrhythmias, in almost all studies, follow-up duration for the ECC was shorter compared with the ILT.^8–10^ One of the unique aspects of the paper in question is that they have reported the longest followed cohort with the ECC, and their work appears to show that this modification is associated with an incidence of late-onset tachyarrhythmia comparable with the ILT.^8,9^ While this is somewhat disappointing, it also indicates that we do not fully understand the causes of tachyarrhythmias in the Fontan population.

## Why are arrhythmias so important in this population?

Atrial tachyarrhythmias are a leading source of morbidity in patients with Fontan surgery.^11^ An initial episode of tachycardia often heralds a pattern of progression towards more frequent and prolonged recurrences.^12^ Unlike patients with a biventricular heart, patients with Fontan physiology often cannot tolerate tachyarrhythmias and experience ventricular dysfunction, heart failure, stroke, and even death.^2^ Carins *et al.*^13^ showed that the onset of arrhythmia (both bradyarrhythmias and tachyarrhythmias) is associated with accelerated progression to heart failure.

There are few studies on the impact of bradyarrhythmias on outcome other than the paper from Carins *et al.*^13^ Unfortunately, the long-term bradyarrhythmias they reported were a combination of sinus node dysfunction (SND), atrioventricular (AV) block, and ‘other’ uncategorized bradyarrhythmia.^13^ It is also unclear whether appropriate management of bradyarrhythmias with pacing can avert or delay the development of cardiac dysfunction. The definition of SND in the present study was a sinus rate < 40 or a junctional escape rate < 40 b.p.m.^6^ This is a stringent definition. The advantage of such a stringent definition is that we can be certain that all patients had severe SND. However, it also means that we do not know whether patients with lesser degrees of dysfunction were missed, and in the process, the opportunity to alter their long-term outcome with appropriate pacing was also missed. We have long known that AV synchrony is vital to maximizing cardiac output in the fragile Fontan population. Sinus node dysfunction with concomitant junctional rhythm is frequently observed among Fontan patients and is associated with a significant decrease in cardiac output, a contributor to heart failure.^14^ Optimal timing of pacing in this population remains a question we must try to answer. Pacemaker implantation in the modern ECC Fontan population is not a simple undertaking, requiring an epicardial device and a repeat sternotomy, often the patient’s third or fourth, or a thoracotomy.^15^

Other forms of Fontan surgery such as the original atriopulmonary connection and the ILT typically allow transvenous atrial pacing for bradyarrhythmias and catheter access for ablation of recalcitrant arrhythmias, either directly or via fenestrations, baffle leaks, or intracardiac trans-baffle punctures.^16^ By bypassing the heart, the ECC Fontan introduces a layer of complexity to managing this most common, highly morbid, and predictable complication. This also complicates the management of atrial tachyarrhythmias by ablation as trans-baffle puncture is required.

Yet, another important consideration of the current paper is our inability to ascertain whether pacing was associated with poorer outcomes. Studies have shown that ventricular pacing is detrimental to the Fontan patient.^17–20^ The Bambino Gesu group report that their preferred method of pacing is DDD (rather than AAI) even in those with isolated SND and report that they were paced for a variable percentage of time in the atrium and 100% of the time in the ventricle. It would be important to look at the clinical outcomes in these ventricular paced patients compared with patients who were not paced in the ventricle. Traditional ventricular pacing can be associated with the development of pacing-induced cardiomyopathy, heart failure, and increased mortality in patients with and without congenital heart disease.^21^ The risk of cardiomyopathy increases with a pacing burden >20% and underlying ventricular dysfunction, the latter being a common finding in congenital heart disease.^22–26^ Despite technological advancements to avoid or treat this using cardiac resynchronization therapy or conduction system pacing (His bundle pacing and left bundle area pacing), the Fontan population is anatomically difficult or impossible to manage with these technologies.^27^

Although initially performed for the palliation of tricuspid atresia, where the systemic ventricle is a morphologic left ventricle, the Fontan procedure is now used on a broader and often more complex population.^26^ As one might expect, the outcomes of this broad spectrum of patients with a single ventricle physiology will also vary haemodynamically and from an arrhythmia standpoint. Expanding indications for the Fontan procedure have resulted in a growing population of patients with a dominant systemic right ventricle, less able to cope with long-term demands of systemic pressures.^26^ The most sizable of this population are those with hypoplastic left heart syndrome and its variants. Of interest, the US Centers for Disease Control and Prevention reports the occurrence of this lesion at 1/3800 neonates, far more common than the lesion for which this procedure was created, tricuspid atresia with an occurrence of 1/9700 neonates.^28^ When considering the incidence of ‘complex’ ventricular arrhythmias, Di Mambro *et al.*^6^ show that patients with a morphological right ventricle have a higher incidence than those with a single ventricle of left ventricle morphology. This is an important and new finding which needs to be corroborated. Lethal arrhythmias (presumably ventricular tachycardia and fibrillation) causing sudden death is an important mode of death in Fontan patients, and their cause has remained elusive.^29^ Perhaps, this paper starts to shed some light on factors that may influence it.

Yet, another consideration is the frequency of arrhythmia surveillance. The Bambino Gesu group reports that they perform electrocardiograms (ECGs) and ambulatory monitors every 3–6 months. This is a degree of surveillance which is enviable. Most centres report ambulatory monitors done much less frequently.^30^ While this may have detected more arrhythmias compared with other centres, it would argue that most other centres are performing arrhythmia surveillance less optimally than required.

In 2012, Khairy *et al.*^16^ called for an assessment of the cohort presented in this issue of *Europace*, the first and oldest patients with ECC Fontan.^6^ Khairy *et al.* challenged us to critically assess the current state of knowledge and determine if the purported advantages of the commonly performed ECC Fontan were real or largely theoretical. We now have empiric clinical evidence. Incidentally, Khairy *et al.* set the bar quite high at perfection since, if this modification fails to eliminate arrhythmias, it confounds their management. While perfection may remain elusive forever, we have cause for optimism based on the data present in this issue of *Europace*. The tachyarrhythmias seen in the ECC Fontan occur later in the life of the ECC Fontan patient and ensure a better quality of life for longer, possibly allowing advancements in medication-, device-, and catheter-based therapies.
